# Economic gradients of health insurance coverage in India

**DOI:** 10.3389/fpubh.2026.1872163

**Published:** 2026-08-13

**Authors:** Puja Pal, Md. Juel Rana

**Affiliations:** 1 V. V. Giri National Labour Institute, Noida, Uttar Pradesh, India; 2 G. B. Pant Social Science Institute (A Constituent Institute of the University of Allahabad), Prayagraj, Uttar Pradesh, India

**Keywords:** expenditure, financial risk protection, healthcare, PMJAY, RSBY, universal health coverage

## Abstract

**Introduction:**

Universal Health Coverage (UHC) aims to ensure accessible, affordable, and quality healthcare for all. In India, achieving UHC is challenging due to very low public health expenditure and a highly privatized healthcare system. This places a significant financial burden on households, often pushing them into poverty and catastrophic health spending. Health insurance is a key tool for reducing financial risk and improving access to healthcare. The study attempts to examine the economic determinants of health insurance coverage in India. It also assesses the temporal-economic gradients of health insurance coverage.

**Methods:**

The National Family Health Survey (NFHS) rounds conducted during 2005–06, 2015–16, and 2019–21 were used. Overall health insurance coverage and different types of health insurance coverage were estimated by the two main economic variables namely wealth quintile and Below Poverty Line (BPL) status. The logistic regression models were applied to estimate the adjusted odds ratio of health insurance coverage by the socioeconomic variables.

**Results and discussion:**

There has been notable progress in enhancing insurance coverage, particularly among poor, primarily through Government-sponsored health insurance (GSHI) schemes. However, a considerable proportion of impoverished households (64 percent of poorest and 58 percent of poorer households) remains without any insurance coverage, largely stemming from a lack of awareness and issues of identification of target groups. Conversely, a notable portion of the middle (71 percent) and affluent classes (70 percent) are accessing the benefits of the GSHI scheme compared to 54 percent among poorest households, mainly due to leakage issues. Consequently, there is a need to recalibrate the exclusion of the middle and affluent classes and the inclusion of the poorest within existing GSHI schemes like PM-JAY. There is need to implement appropriate strategies such as linking SECC data with Aadhaar, conducting regular enrolment audits, developing state-level targeting metrics, and using community-based enrolment to reduce identification errors and leakage. These should be paired with broader reforms, including integrating state and central schemes, expanding outpatient coverage and awareness, regulating private providers, and strengthening primary healthcare alongside insurance expansion.

## Introduction

Accessible, affordable, and quality healthcare services to all is the basic right of an individual. Towards this objective, the goal of ‘Universal Health Coverage’ (UHC) holds central importance. In 2012, the United Nations (UN) adopted a formal resolution on UHC (1). The Sustainable Development Goals (SDGs) added global momentum in 2015 by including UHC as target 3.8. which focuses on the accessibility of safe, quality, effective, and affordable essential healthcare services, medicines, and vaccines to all (2). The UHC has three critical dimensions including coverage, benefits, and financial risk, this study particularly focuses on the first critical dimension, i.e., coverage of health insurance (1).

India spends only about 1.5 percent of its GDP which is well below the global average of 6 per cent which is one of the lowest in the world (3). The consistently low public expenditure on healthcare has resulted in two-thirds of the population seeking costlier treatments in the private sector which lies in the out-of-pocket healthcare expenditure (4). India has one of the most highly privatized healthcare systems in the world. Among South Asian countries, India ranks 3rd on Out-of-Pocket Expenditure (OOPE) as a share of total health expenditure (5). The high OOPE (around 54.7 percent of total health expenditure) has become one of the primary sources of financing health care services in India which adversely impacts individuals pushing them into poverty and a debt trap (6–8). The accelerated OOPE has resulted in a huge financial burden to the people in general and the poorest in particular. Due to high OOPE around 3.3 per cent of the population was pushed into poverty and some 16.5 percent of the population faced catastrophic health expenditure (9, 10).

The consistently low public expenditure and high OOPE are a matter of concern in the Indian healthcare sector (11). Against this backdrop, health insurance is one of the important instruments to achieve the UHC goal, as it provides financial security and safety to individuals against healthcare costs. Health insurance can be broadly seen as a risk-pooling arrangement that helps to reduce or avoid healthcare expenses for an individual (1). It mitigates the financial risk related to healthcare. Another important dimension of health insurance is its multidimensional social security measure which provides health protection to the population.

India’s early initiative towards providing health insurance can be traced seven decades back when two central government schemes namely Employees State Insurance Schemes (ESIS) in 1952 and the Central Government Health Scheme (CGHS) in 1954 were constituted. However, both these schemes only catered to the needs of a very small proportion of India’s population who are central government employees or employed in the formal sector. The health insurance sector was nationalized in 1972 when around 107 private companies were brought under the General Insurance Corporation (GIC). Then in the post-economic reform period (after 1991), the government allowed private and foreign companies to enter the insurance market with the enactment of the Insurance Regulatory and Development Act (IRDA) in 1999 (12). The privatization of the health insurance sector in India led to the flourishment of the insurance market particularly among the urban middle-class population (13). However, in the unregulated environment, the private health insurance market became so costly that even the affluent upper middle class could not afford it. Thus, all these factors led to a huge unmet need and demand for health insurance which influenced the government to extend the coverage under Government Subsidized Health Insurance schemes in the last one and a half decades.

The ‘Rashtriya Swasthya Bima Yojana (RSBY)’ was the first central government-sponsored insurance scheme implemented in 2008. The RSBY targeted families in the Below Poverty Line (BPL) category and provided financial coverage of Rs 30,000 per family for most diseases that require hospitalization and the coverage was extended to five members of the family (14). Many studies pointed to the failure of RSBY due to factors like low coverage, poor implementation, fraud, bogus beneficiaries, exclusion of vulnerable individuals, and lack of awareness (15, 16). Among the beneficiaries, the hospitalization rate under RSBY was also quite low, i.e., 1 percent as compared to the national average, i.e., 2.6 percent (16). Despite its several failures, the expansion of coverage under RSBY was unparalleled to the coverage by any other scheme. Another important role played by RSBY is that it paved the way for many states to include added benefits to the RSBY scheme or increase the population coverage while many other states started their state health insurance schemes (17). During the period 2008–2018, many state governments started a state-specific health insurance scheme to target poor and vulnerable populations. For example, West Bengal launched the ‘West Bengal Health Scheme’ in 2008 which later in 2014 got revamped to ‘West Bengal Health for All Employees and Pensioners Cashless Medical Treatment Scheme’. Similarly, Tamil Nadu introduced ‘Chief Minister Kalaignar Insurance Scheme for Life-Saving Treatments’ in 2009; Kerala started ‘Comprehensive Health Insurance Scheme’ in 2008; Maharashtra announced ‘Rajiv Gandhi Jeevandayee Aarogya Yojana’ in 2010.

To overcome the limitations of the RSBY and to reduce the OOPE, the government of India introduced the ‘Pradhan Mantri Jan Arogya Yojana’ (PM-JAY) in 2018. It is another major turning point in the Indian insurance landscape as it is one of the largest health insurance schemes in the world. This is a noteworthy step towards the goal of achieving UHC in India. It aims to target the poorest 40 percent of the total population which will approximately cover 500 million people in India. The financial coverage under PM-JAY increased 16 times to Rs 5 lakh per family. Unlike RSBY, it covers all family members for secondary and tertiary healthcare hospitalizations. Given certain criticism of RSBY for failing to deliver its targeted goal, PM-JAY has given new hope in terms of expansion of coverage and protecting poor and vulnerable populations from catastrophic health expenditures (18). However, the effectiveness of the programme is yet to be evaluated.

In the last one and a half decades, the central and the state governments have implemented target-based health insurance schemes as mentioned above. All these schemes target the poor and vulnerable as they tend to spend a larger proportion of their income on health care compared to their richer counterparts (19, 20). Over the period of time, the share of GSHI like RSBY, several state-sponsored health insurances, and later PM-JAY has increased manifold times in the overall health financing in India which particularly targets poor and vulnerable groups (21). However, little is known about how groups of people with different levels of economic status have benefitted from these health insurance schemes. Existing evaluations have largely examined RSBY or PM-JAY individually and at a single point in time; unlike these studies, the present analysis pools three rounds of nationally representative data spanning the pre-RSBY, RSBY, and PM-JAY eras (2005–06 to 2019–21) to characterise the economic gradient in coverage and how it has moved over time, and further decomposes coverage by insurance type (SHI, GSHI, PHI, and ‘other’) in the latest round.

Evidence from other low and middle income countries with respect to coverage of health insurance could provide useful comparative context for understanding India’ experience. Countries in Sub-Saharan Africa (SSA) are increasingly adopting the public contributory health insurance approach as a mechanism to provide financial protection to the population. However, a study by Barasa et al. (22) assessed the level of inequality in health insurance coverage for 36 SSA countries and found that coverage remained low and proportionally concentrated among the richer and more educated households.

Although UHC covers broader dimensions, including population coverage, quality of care, equity, benefit packages, and financial risk protection, this study focuses specifically on one of its key components: health insurance coverage. In addition, while inequities in healthcare outcomes remain a major challenge towards achieving UHC, this study examines the economic aspects of health insurance coverage in India. A large number of studies have documented socioeconomic gradients in healthcare, demonstrating that healthcare outcomes and health-related behaviours are systematically patterned by socioeconomic status across the population (23). In this study, the term ‘economic gradient’ refers to the systematic and monotonic variation in health insurance coverage across the entire distribution of household wealth status from the poorest to the richest wealth quintiles, as well as by the status of Below Poverty Line (BPL). Thus, the analysis goes beyond a simple binary comparison between poor and non-poor households to examine how insurance coverage changes progressively across different economic groups.

Thus, the study firstly examines the economic determinants of health insurance coverage in India. Secondly, it assesses the temporal-economic gradients of health insurance coverage in India. Thirdly, it assesses the economic determinants of types of health insurance in India.

## Data and methods

### Data and sample

The study used the latest three rounds of the National Family Health Survey (NFHS) conducted during 2005–06, 2015–16, and 2019–21 (24–26). The NFHS estimates are comparable to those from the Demographic and Health Surveys (DHS), which are conducted in over 90 countries worldwide. The NFHS was initiated in the early 1990s, and its fifth round was conducted during 2019–21. The NFHS is a large-scale survey providing reliable and consistent data on vital indicators of demographic, health, and nutrition, like reproductive and child health, fertility, family planning, nutrition, non-communicable diseases, health insurance, and many other related issues. The NFHS-5 (2019–21) provides information for 6,36,699 households covering 707 districts from 29 States and 7 Union Territories. The NFHS-4 (2015–16) covered 601,509 households from 640 districts of 29 States and 7 Union Territories in India. The NFHS-3 (2005–06) has 109,041 sample households, covering 99 percent of India’s population across all 29 states.

### Variables

The study’s dependent variable is health insurance coverage, which is binary. If any member of a household is covered by any health insurance scheme, it is coded as 1; otherwise, it is coded as 0. The predictor variables include economic characteristics of households, namely wealth status and BPL status. The wealth status of the household is constructed using a comprehensive methodology where households are equally divided into five categories with 20 percent of the households based on the number and kind of assets and amenities they own, including television bicycle, or car, and housing characteristics like drinking water, toilet facilities, and flooring materials, etc. (26). Based on this wealth status, the households are categorized into five quintiles such as poorest, poorer, middle, richer, and richest. In the case of a BPL household, if the household possesses a BPL card, it is coded as 1, it is coded as 0. Other independent variables are the level of education (no education, primary, secondary, and higher), place of residence (rural and urban), caste (scheduled caste (SC), scheduled tribe (ST), other backward caste (OBC), and others), and religion (Hindu, Muslim, Christian, and others), and state.

The NITI Aayog (2021) in its latest report on ‘India’s Missing Middle’ categorized India’s existing health insurance schemes broadly into three categories (based on the financing sources) *viz.* (1) government subsidized schemes which include RSBY and state health insurance schemes, (2) social health insurance schemes (includes ESIS and CGHS), and (3) private voluntary schemes comprising of community health insurance programme, other health insurance through an employer, medical reimbursement from employer and other privately purchased commercial health insurance. Based on NITI Aayog’s (2021) classification, for the analyses of type of health insurance coverage, it is categorized into four categories particularly (1) coverage of health insurance in Social Health Insurance (SHI) scheme, (2) coverage of health insurance under Government Subsidized Health Insurance (GSHI) scheme, (3) coverage of health insurance under Private Health Insurance (PHI) schemes and (4) coverage of health insurance under other health insurance scheme (Others) (3). Each of these variables is binary, coded as 1 if yes and 0 otherwise.

### Statistical analysis

The study carries out univariate, bivariate, and multivariate analyses to assess the predictors of health insurance coverage in India. The univariate analysis presents the percentage distribution of the sample across the categories of each study variable. The bivariate analysis examined the percentage of health insurance coverage by the socioeconomic characteristics of the sampled households.

The unadjusted and adjusted predicted probabilities (percentages) of health insurance were computed from the logistic regression models. Two sets of logistic regression models were applied to examine the socioeconomic determinants of health insurance coverage in India. One set of regression models is applied to the latest NFHS sample, while another set is applied to the pooled sample combining the latest three NFHS rounds. In the pooled sample, interaction analyses were conducted to examine the mediating effects of time on the relationship between economic status and health insurance coverage in India. The interaction analyses were conducted for two economic indicators: household wealth quintiles and BPL status. Two more logistic regression models were applied to the pooled sample of the latest three NFHS rounds to avoid multicollinearity arising from the intersection models. The variance inflation factors (VIFs) were estimated for these models to assess multicollinearity and are presented in Appendices 1, 2.

The percentages of the four types of health insurance schemes, namely SHI, GSHI, PHI, and ‘others’, are estimated by categories of economic variables to understand the nature of health insurance coverage across these factors. All statistical analyses are conducted in Stata version 16. For the bivariate and multivariate analyses, the national weight with the complex survey design (*svyset*) was applied.

## Results

### Sample characteristics

**Figure 1:**
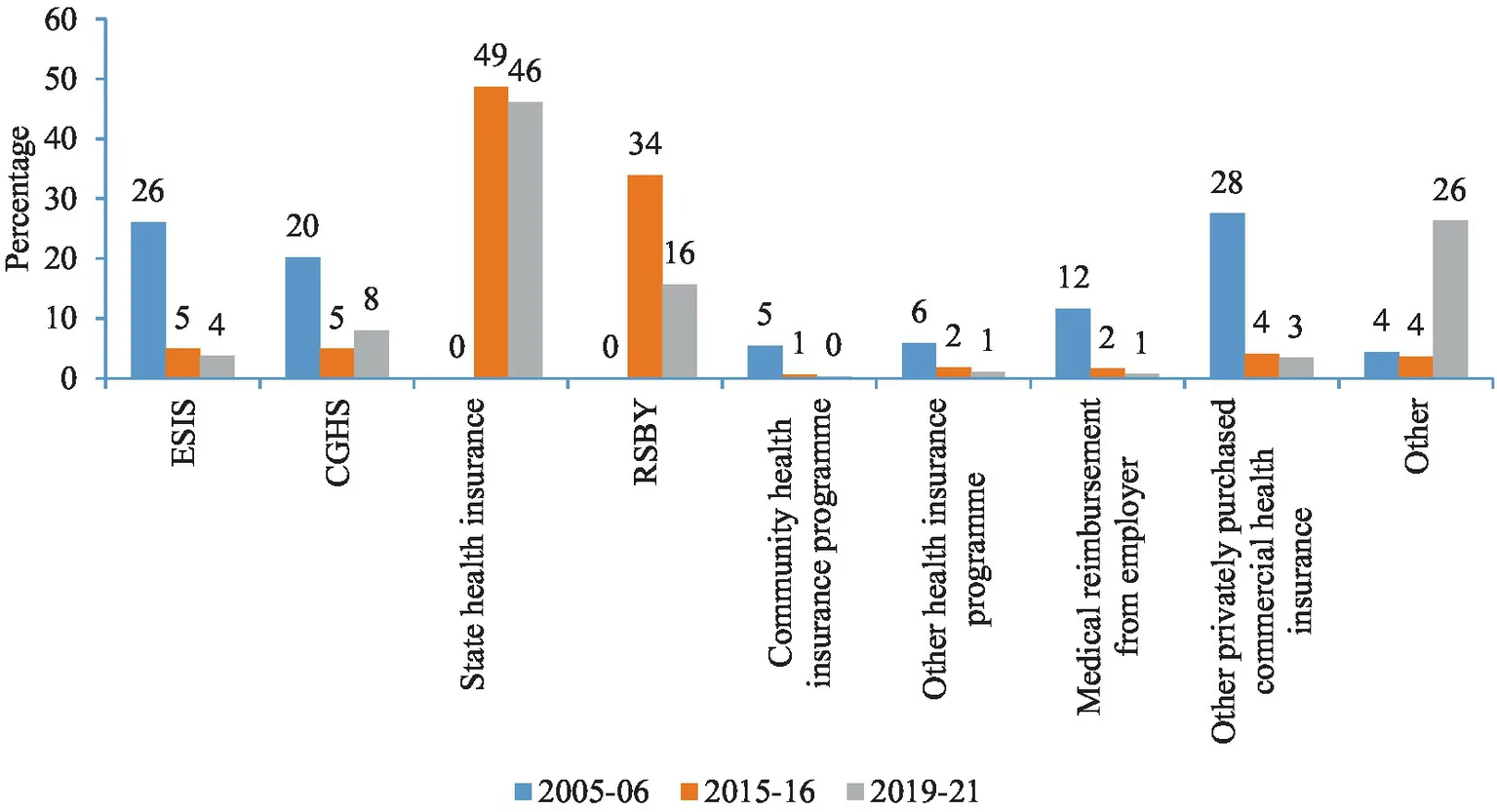
Types of health insurance schemes in India during 2004–05, 2015–16, and 2019–21.

The socioeconomic characteristics of the sample are presented in Table 1. During 2019–21, around 42 percent of households reported having at least one member covered by health insurance, compared with 26 percent in 2015–16 and only 6 percent in 2005–06. Types of health insurance coverage during 2005–06, 2015–16, and 2019–21 are presented in Figure 1. During 2005–06, the largest share of health insurance was contributed by the privately purchased health insurance scheme (28 percent), ESIS (26 percent), and CGHS (20 percent). After 10 years, during 2015–16, the major share of coverage is from the state health insurance scheme (49 percent) and RSBY (34 percent). After a few years, from 2019–21, the share of the state health insurance scheme (46 percent) and RSBY (16 percent) remains high, while the share of insurance by ‘Others’ stands at one-fourth of total health insurance coverage (26 percent). We categorized health insurance schemes into four groups: SHI, GSHI, PHI, and ‘Others’. It is observed that GSHI has the predominant form of insurance coverage with 61.8 percent during 2019–21. This is followed by the coverage in the ‘Others’ category, which has increased significantly to 26 percent during 2019–21 from 4 percent during 2015–16. The share of SHI and PHI is 12 percent and 5 percent, respectively, during 2019–21.

**Table 1:** Sample distribution of study variables, NFHS (2005–06, 2015–16 and 2019–21).

Background characteristics	NFHS-3 (2005–06)	NFHS-4 (2015–16)	NFHS-5 (2019–21)
Health insurance			
No	93.7 (93.6, 93.8)	74.1 (74, 74.3)	57.4 (57.3, 57.5)
Yes	6.3 (6.2, 6.4)	25.9 (25.7, 26)	42.6 (42.5, 42.7)
Wealth quintile			
Poorest	13.4 (13.2, 13.6)	21.9 (21.8, 22)	23.4 (23.3, 23.5)
Poorer	15.2 (15, 15.4)	21.6 (21.5, 21.7)	22.2 (22.1, 22.3)
Middle	19.2 (19, 19.4)	20.3 (20.2, 20.4)	20.3 (20.2, 20.4)
Richer	23.4 (23.1, 23.6)	18.7 (18.6, 18.8)	18.1 (18, 18.2)
Richest	28.8 (28.5, 29)	17.5 (17.4, 17.6)	16.1 (16, 16.2)
BPL card			
No	78.1 (77.9, 78.4)	61.8 (61.7, 61.9)	51.3 (51.2, 51.4)
Yes	21.9 (21.6, 22.1)	38.2 (38.1, 38.3)	48.7 (48.6, 48.8)
Place of residence			
Urban	46.1 (45.8, 46.4)	29.3 (29.1, 29.4)	25.2 (25, 25.3)
Rural	53.9 (53.6, 54.2)	70.7 (70.6, 70.9)	74.8 (74.7, 75)
Level of education			
No education preschool	31.3 (31, 31.6)	31.4 (31.3, 31.6)	29.8 (29.7, 29.9)
Primary	18 (17.7, 18.2)	18.6 (18.5, 18.7)	18.5 (18.4, 18.6)
Secondary	38.7 (38.4, 39)	40.7 (40.6, 40.8)	42.3 (42.1, 42.4)
Higher	12 (11.9, 12.2)	9.2 (9.2, 9.3)	9.4 (9.4, 9.5)
Caste			
SC	16.7 (16.5, 17)	18 (17.9, 18.1)	19.3 (19.2, 19.4)
ST	13.5 (13.3, 13.7)	19 (18.9, 19.1)	19.4 (19.3, 19.5)
OBC	31.6 (31.3, 31.8)	37.6 (37.5, 37.7)	36.7 (36.6, 36.9)
Others	38.2 (37.9, 38.5)	25.4 (25.3, 25.5)	24.6 (24.4, 24.7)
Religion			
Hindu	73.4 (73.1, 73.6)	74.5 (74.4, 74.7)	75.7 (75.6, 75.8)
Muslim	12.2 (12.1, 12.4)	12.1 (12.1, 12.2)	11.4 (11.3, 11.5)
Christian	9.2 (9, 9.4)	8.2 (8.1, 8.2)	7.8 (7.7, 7.9)
Others	5.2 (5, 5.3)	5.1 (5.1, 5.2)	5.1 (5, 5.1)
Sample Size	1,09,041	6,36,699	6,01,509

Sample size is unweighted.

During 2019–21, the share of the selected sample in the analyses of our study is highest from the poorest wealth quintile (23.4 percent), followed by the poorer (22.2 percent), middle (20.3 percent), richer (18.1 percent), and richest (16.1percent) (Table 1). Around 48.7 percent of the sampled households had BPL cards. About one-fourth of households (25.2 percent) are in urban areas. Out of the sampled households, around 42.3 percent of household heads had secondary education, followed by no education (29.8 percent), primary education (18.5 percent), and higher education (9.4 percent). The OBC groups had the highest share (36.7 percent), followed by the ‘Other’ category (24.6 percent), ST (19.4 percent), and SC (19.3 percent). The largest share of sampled households was Hindu (75.7 percent), followed by Muslim (11.4 percent), Christian (7.8 percent), and Others (5.1 percent) during 2019–21.

### Economic determinants of health insurance coverage

Table 2 (Column 4) shows the percentage of households with health insurance coverage by economic characteristics, namely wealth status (quintiles) and BPL status. During 2019–21, health insurance coverage was highest among middle households (44.1 percent) and lowest among the poorest households (36.1 percent), while the richer and poorer households had similar levels of coverage (~42 percent). The richest households have relatively lower insurance coverage (40.8 percent) than their richer counterpart. The coverage of health insurance among BPL households is considerably higher (48 percent) than in non-BPL households (35 percent). The percentage of health insurance coverage by different social factors is presented in Appendix Table 3.

**Table 2:** Percentage of households with health insurance coverage by economic characteristics in India, 2005–06, 2015–16, and 2019–21.

Economic variables	NFHS-3 (2005–06)	NFHS-4 (2015–16)	NFHS-5 (2019–21)
Wealth quintile			
Poorest	0.1 (0.1, 0.2)	21.6 (21.1, 22)	36.1 (35.6, 36.6)
Poorer	0.7 (0.5, 0.8)	28.4 (28, 28.9)	42 (41.6, 42.5)
Middle	2.2 (1.9, 2.6)	32.3 (31.8, 32.8)	44.1 (43.6, 44.6)
Richer	5.1 (4.5, 5.7)	30.6 (30.1, 31.2)	42.2 (41.6, 42.7)
Richest	16.4 (15.2, 17.7)	30.5 (29.8, 31.3)	40.8 (40.1, 41.4)
BPL card			
Yes	6 (5.5, 6.4)	21.9 (21.5, 22.2)	35 (34.6, 35.4)
No	2 (1.7, 2.3)	39.5 (39.1, 39.9)	48.3 (48, 48.6)
Total	4.9 (4.5, 5.3)	28.7 (28.3, 29)	41 (40.7, 41.3)

National Family Health Surveys; Estimates are weighted applying the complex survey design.

Table 3 provides unadjusted and adjusted predicted prevalence of health insurance coverage by economic characteristics, namely wealth and BPL status, using the pooled sample and the recent sample (NFHS-5). For the NFHS-5, the findings show that the adjusted predicted prevalence of coverage rises with households’ wealth status after controlling for the effects of other social factors. The predicted prevalence of coverage is highest among the richest households (46.5 percent), followed by richer (41.3 percent), middle (40.6 percent), and poorer (40 percent) compared to the poorest households (37.2 percent). Thus, the adjusted model shows a consistent upward gradient with wealth, once other factors are held constant. Contrary to the results for wealth status, the adjusted predicted prevalence of households with BPL cards at 47.3 percent which is far more compared with 35 percent among non-BPL households (see Appendix Tables 4, 5).

**Table 3:** Unadjusted and adjusted predicted prevalence of health insurance coverage by background characteristics in India, NFHS-3 (2005–06), NFHS-4 (2015–16), and NFHS-5 (2019–21).

Background characteristics	Pooled sample (NFHS-3, 4 and 5)				NFHS-5 (2019–21)	
	Unadjusted	Adjusted (M1)	Adjusted (M2)	Adjusted (M3)	Unadjusted	Adjusted
Survey rounds						
2005–06 (Ref.)	4.9 (4.5, 5.3)	4.8 (4.5, 5.2)	5.5 (5.1, 5.9)	–	–	–
2015–16	28.7 (28.3, 29)	29.1 (28.8, 29.5)	29 (28.7, 29.4)	–	–	–
2019–21	41 (40.7, 41.3)	40.3 (40, 40.7)	40.2 (39.8, 40.5)	–	–	–
Wealth quintile						
Poorest (Ref.)	26.8 (26.4, 27.1)	22.9 (22.6, 23.2)	22.9 (22.6, 23.2)	–	36.1 (35.6, 36.6)	37.2 (36.7, 37.6)
Poorer	32.7 (32.3, 33)	29.9 (29.6, 30.2)	29.9 (29.6, 30.2)	–	42 (41.6, 42.5)	40 (39.6, 40.4)
Middle	35.5 (35.1, 35.8)	34.4 (34, 34.7)	34.3 (34, 34.7)	–	44.1 (43.6, 44.6)	40.6 (40.2, 41)
Richer	34 (33.6, 34.4)	36.3 (36, 36.7)	36.3 (35.9, 36.6)	–	42.2 (41.6, 42.7)	41.3 (40.9, 41.7)
Richest	34.2 (33.7, 34.6)	41.7 (41.2, 42.2)	41.5 (41, 42)	–	40.8 (40.1, 41.4)	46.5 (45.9, 47.2)
Below poverty line						
No (Ref.)	26 (25.8, 26.3)	26.3 (26.1, 26.5)	26.2 (26, 26.4)	–	35 (34.6, 35.4)	35.6 (35.3, 35.9)
Yes	42.1 (41.8, 42.4)	41.7 (41.4, 42)	41.6 (41.3, 41.9)	–	48.3 (48, 48.6)	47.3 (47, 47.7)
Interaction between survey rounds and wealth quintile						
2005–06 and Poorest (Ref.)	0.1 (0.1, 0.2)	0.1 (0.1, 0.2)	–	0.1 (0.1, 0.2)	–	–
2005–06 and Poorer	0.7 (0.5, 0.8)	0.6 (0.5, 0.7)	–	0.6 (0.5, 0.7)	–	–
2005–06 and Middle	2.2 (1.9, 2.6)	2.1 (1.8, 2.5)	–	2.1 (1.8, 2.5)	–	–
2005–06 and Richer	5.1 (4.5, 5.7)	5.2 (4.6, 5.8)	–	5.2 (4.6, 5.8)	–	–
2005–06 and Richest	16.4 (15.2, 17.7)	17.7 (16.3, 19.1)	–	17.7 (16.3, 19.1)	–	–
2015–16 and Poorest	21.6 (21.1, 22.1)	17.8 (17.4, 18.3)	–	17.8 (17.4, 18.3)	–	–
2015–16 and Poorer	28.4 (27.9, 29)	25.8 (25.3, 26.3)	–	25.8 (25.3, 26.3)	–	–
2015–16 and Middle	32.3 (31.7, 33)	31.6 (31.1, 32.2)	–	31.6 (31.1, 32.2)	–	–
2015–16 and Richer	30.6 (30, 31.2)	34.1 (33.5, 34.6)	–	34.1 (33.5, 34.6)	–	–
2015–16 and Richest	30.5 (29.8, 31.3)	40 (39.1, 40.9)	–	40 (39.1, 40.9)	–	–
2019–21 and Poorest	36.1 (35.6, 36.6)	31.3 (30.8, 31.8)	–	31.3 (30.8, 31.8)	–	–
2019–21 and Poorer	42 (41.5, 42.6)	38.5 (38, 39)	–	38.5 (38, 39)	–	–
2019–21 and Middle	44.1 (43.5, 44.7)	42.2 (41.7, 42.8)	–	42.2 (41.7, 42.8)	–	–
2019–21 and Richer	42.2 (41.6, 42.7)	43.6 (43, 44.1)	–	43.6 (43, 44.1)	–	–
2019–21 and Richest	40.8 (40.1, 41.4)	47.2 (46.6, 47.9)	–	47.2 (46.6, 47.9)	–	–
Interaction between survey rounds and BPL						
2005–06 and No BPL (Ref.)	6 (5.5, 6.4)	5.1 (4.8, 5.5)	–	5.1 (4.8, 5.5)	–	–
2005–06 and BPL	2 (1.7, 2.3)	4.1 (3.6, 4.6)	–	4.1 (3.6, 4.6)	–	–
2015–16 and No BPL	21.9 (21.5, 22.2)	21.1 (20.8, 21.5)	–	21.1 (20.8, 21.5)	–	-
2015–16 and BPL	39.5 (39, 40.1)	41.3 (40.7, 41.8)	–	41.3 (40.7, 41.8)	–	–
2019–21 and No BPL	35 (34.6, 35.4)	34.7 (34.3, 35.2)	–	34.7 (34.3, 35.2)	–	–
2019–21 and BPL	48.3 (47.9, 48.7)	48.5 (48.1, 48.9)	–	48.5 (48.1, 48.9)	–	–
Number of observations	–	13,47,249	13,47,249	13,47,249	–	6,36,699
Number of Strata	–	3,585	3,585	3,585	–	2,710
Number of PSUs	–	62,544	62,544	62,544	–	30,170
*F* statistic	–	698***	1146***	698***	–	632***

CI stands for 95% Confidence Interval; ***p < 0.001; the *p*-value of each category of the independent variables is <=0.001; Ref. stands for Reference group in the variable; Level of education, place of residence, caste, and religion, and state are included in both the logistic regression model.

### Temporal economic gradients of health insurance coverage

Table 2 presents the percentage of health insurance coverage for the years 2005–06, 2015–16, and 2019–21 separately. The findings show a quantum leap in the past decade, with 41 percent of surveyed households having at least one member covered under any health insurance scheme during 2019–21, up from only 4.9 percent during 2005–06. The prevalence is lowest among the poorest households (36.1 percent), and it is highest among the middle households (44.1 percent) during 2019–21. A similar pattern was observed 5 years back in 2015–16. Ten years back, the prevalence increased monotonically as the wealth status of the households improved during 2005–06. The percentage of households covered with health insurance shows a rise among the households in each of the wealth quintiles from 2005–06 to 2019–21, but the rate of progress is considerably higher among the poorest, poorer, and middle households as compared to the richer and richest households. On the other hand, during 2019–21, the coverage of health insurance among BPL households is higher (48.3 percent) than non-BPL households (35 percent). Interestingly, the coverage among BPL households was lower (2 percent) than non-BPL households (6 percent) in 2005–06. It indicates that among the BPL households, the coverage of health insurance has increased at a faster pace than the non-BPL households.

Table 3 shows adjusted predicted prevalence (M1), which rose from 4.8 percent in 2005–06 to 29.1 percent in 2015–16 to 40.3 percent in the latest round of NFHS. The interaction between NFHS rounds and wealth quintiles suggests that, within each survey rounds, the predicted prevalence of health insurance coverage improves with wealth status of household. During 2015–16, the predicted prevalence of coverage increased from 17.7 percent among the poorest to 34.1 percent among the richest households. A similar pattern is also observed for the sample of NFHS conducted during 2019–21. The interaction between NFHS rounds and BPL households shows that the predicted prevalence of getting health insurance coverage is higher among the BPL households during 2015–16 and 2019–21 as compared to the reference category (combined sample of non-BPL households surveyed in all three rounds and all sample surveyed during 2005–06). Thus, the interaction model shows that BPL households have more likelihood of getting covered under insurance schemes in the recent period of time.

### Economic gradients of types of health insurance coverage

Table 4 presents the percentage share of health insurance schemes among the households in which at least one member is covered with a health insurance policy by economic characteristics during 2005–06, 2015–16, and 2019–21. During 2019–21, the share of GSHI is higher among the middle (71 percent), richer (70 percent), and poorer (65 percent) households than the national average (62 percent), while it is lower among the richest (48 percent) and poorest (54 percent) households. The share of PHI is very high among the richest households (20 percent) compared with the national average (6 percent), while it is quite low among the rest of the households (<5 percent). The share of SHI is relatively higher among the richest (23 percent) and richer (13 percent) households than India’s average (12 percent). The share of ‘other’ health insurance coverage is significantly higher among the poorest (43 percent) and poorer (32 percent) households than the national average (26 percent) during 2019–21. During the same period, among the households with BPL cards, the share of GSHI (65 percent) and ‘other’ (30 percent) health insurance coverage was significantly higher than that of the non-BPL households. At the same time, the share of SHI (16 percent) and PHI (10 percent) is higher among the households without BPL cards than among the households with BPL cards.

**Table 4:** Types of health insurance coverage by economic characteristics during 2004–05, 2015–16, and 2019–21.

Economic characteristics	NFHS-3 (2005–06)				NFHS-4 (2015–16)				NFHS-5 (2019–21)			
	SHI	GSHI	PHI	Others	SHI	GSHI	PHI	Others	SHI	GSHI	PHI	Others
Wealth quintile												
Poorest	52.5	–	42.1	7.4	2.6	97.8	1	2.6	5.6	53.6	0.6	43.4
Poorer	32.4	–	57.4	9.3	3.1	95	1.8	3.2	7.1	64.5	1	31.8
Middle	38.2	–	51.5	9.5	4.9	91.8	2.9	3.3	9.7	71.2	1.9	22.5
Richer	49.8	–	45.5	5.5	10.4	83.4	6.5	3.4	13	69.5	4.5	19.1
Richest	46.7	–	51.5	3.2	25.5	49.7	26.3	5.5	23	48.1	20	16.1
BPL card												
No	48.3	–	49	4	16.7	68.7	14.7	5	15.9	57.9	9.8	22.3
Yes	30.2	–	60.9	8.1	3.7	94.7	2.4	2.5	7.9	65.2	1.9	29.8
Total	46.3	–	50.4	4.4	9.8	82.6	8.1	3.6	11.7	61.8	5.5	26.3

SHI, Social health insurance schemes (ESIS, CGHS); GSH, govt-subsidized health insurance scheme (state health insurance scheme, RSBY); PHI, Private health insurance scheme (community health insurance programme, other health insurance through employer, medical reimbursement from employer, and other privately purchased commercial health insurance), Others: any other health insurance scheme.

## Discussion

Health insurance provides a safety net to the poor population by reducing the financial burden during adverse health shocks. Studies have indicated the important role of health insurance in improving access to better health outcomes (27). However, little evidence is available to suggest that publicly financed health insurance schemes played a substantial role in reducing OOPE in India (28, 29), mainly due to inadequate coverage of outpatient care and medicine costs. Despite this, health insurance has emerged as a prime instrument of health financing reform in India to achieve UHC, especially after the launch of several publicly funded health insurance schemes (18). The coverage of health insurance particularly among poor and vulnerable populations is a major challenge in itself in achieving UHC in India. In this context, the study focuses on examining the economic determinants of health insurance coverage in India.

There are mainly two broad approaches to achieve UHC through health insurance in developing countries. Countries like China, Thailand, Chile, Mexico, Brazil, etc., adopted a universal coverage approach where they extended the coverage to the entire population for the priority package of services. On the other hand, there are countries like the Philippines, Cambodia, Vietnam, Zambia, Ghana, India, etc. adopted a targeted approach where they prioritize specific population groups for comprehensive services (30, 31). Although India has adopted the target-based approach to health insurance coverage, especially the poor and vulnerable section, the relative position of India (42 percent) in terms of health insurance coverage is far away from other developing countries such as South Korea (~100 percent), Thailand (~100 percent), China (96 percent), Vietnam (87 percent), Philippine (89 percent), etc. In this background, the findings from this study would have a significant impact on the strategic implementation of the schemes for providing financial health protections to the poor and vulnerable groups of people in India.

In the last one and half decades, the health insurance coverage in India has increased considerably particularly among the poor households in India. The main attributable factor for this development is providing financial risk protection for healthcare to the poor vulnerable population since the launch of RSBY in 2008 which targeted the poor and vulnerable. Subsequently, similar GSHI schemes were also rolled out in different states of India to date. The PM-JAY as the world’s largest GSHI was implemented in 2018 targeting the 40 percent bottom poor and vulnerable as an improved version of RSBY in several aspects, particularly in terms of much bigger vertical coverage further, the implementation of PM-JAY shows India’s greater commitment to achieve UHC through the GSHI scheme which extends coverage to poor and vulnerable populations. The target-based GSHI like PM-JAY may be an effective option for India as developing countries do not have adequate resources to finance the healthcare needs of their entire population. So, instead of providing universal health care to all, the government focuses on subsidizing the insurance premiums for at least the poor population (32).

Despite significant improvement in coverage among relatively poor households in the last decades, around 64 percent of poorest households and 58 percent of poorer households are not covered by any health insurance during 2019–21. On the same line, more than half of the BPL households (52 percent) are not covered by any health insurance scheme. Thus, the study highlights the inadequate health insurance coverage among poor and vulnerable households. There could be two main reasons behind this. Firstly, a lack of awareness about the provisions of GSHI, like RSBY, PM-JAY, and the procedure for availing the benefits (33). Studies have highlighted that higher enrolment rate of health insurance scheme in region is linked with higher awareness like Karnataka and Chhattisgarh have registered higher enrolment and high awareness (34, 35) while states like Maharashtra reported low coverage with low awareness (36). A study by Parisi et al. (37) also found that awareness of PM-JAY and of one’s own eligibility is itself socio-economically graded, which indicates that marginalized group is less aware. Secondly, the issues in identifying the target groups. Under RSBY and many other state health insurance schemes, the eligibility criteria for the poor use the state BPL lists and PM-JAY uses data from the Socio-Economic and Caste Census (SECC) conducted in 2011. Many studies found the exclusion of the poor and vulnerable groups in the BPL list (38, 39). The one-decade-old SECC data cannot capture changes in households’ economic conditions in the recent past. Thus, addressing data inaccuracy and poorly defined target populations for identification may be crucial to improving health insurance coverage. Though NFHS does not directly measure awareness or administrative targeting processes, these should be read as interpretations consistent with prior evidence rather than findings established directly from the present data.

Among the poorest and poorer groups of households, there is a sharp rise in the ‘other’ health insurance coverage by 41 percent and 29 percent, respectively, from 2015–16 to 2019–21. Perhaps, the launch of PM-JAY in 2018 could have majorly contributed to the sharp rise of health insurance among these poor groups of households. Since the questionnaire of NFHS-5 (2019–21) did not include the option of ‘PM-JAY’ in response to the question on types of health insurance coverage while administering data collection tool, i.e., questionnaire, the households which are covered with the PM-JAY were categorized in ‘other’ category. Thus, it may be safe to consider that the major part of the ‘other’ type of health insurance is covered by PM-JAY. Since this scheme has targeted the poor and vulnerable, a noticeable increase in coverage has been registered. A study by Mohanty et al. (40) reported that introduction of PM-JAY coincided with increased public health insurance coverage among the poorest wealth quintiles. However, the improvement could not be only attributed to PM-JAY, other possible reasons could be expanded coverage in other schemes like community health insurance, or from broader demand-side changes.

The coverage of health insurance in general and coverage of GSHI, in particular, are highest among households with middle-wealth status. This reality contradicts the claims from other earlier studies. A study using the data from the 75th round of the National Sample Survey conducted during 2017–18 showed that around 50 percent of the population who are neither deprived nor affluent are devoid of any kind of financial health protection (41). The NITI Aayog Report (2021) estimates at least 30 percent of India’s population is ‘missing middle’ which is devoid of any kind of financial health protection. This uncovered population is neither poor nor rich as per the report. The paradox of the simultaneous presence of ‘missing middle’ in the existing study and the highest health insurance coverage in the middle households in the present can be disentangled if it is defined concurrently (3). In this study, the middle class is defined based on the wealth quintiles while the consumer expenditure is used as the criteria in other studies. Therefore, further in-depth investigation is needed to know who are those missing middle and what are their economic characteristics.

The share of GSHI is considerably higher among households in middle wealth status as compared to the poorest and poorer wealth quintiles. Even, among the richest households, about half of the insurance is covered by the GSHI which is mainly meant for the poorest groups of society. The findings indicate the leakage problem which normally exists in target-based welfare schemes. The presence of substantial leakages in the GSHI scheme results in the exclusion of benefits to poor and vulnerable individuals (we note that this leakage is inferred descriptively from that fact that substantial share of GSHI coverage accrues to the non-poor groups who are not the intended beneficiaries of the schemes, rather than directly measured through beneficiary-level audit or administrative verification). Many studies have identified the ‘leakage issue’ as crucial factor behind the underperformance of targeted health insurance schemes (30, 36, 42). A study by Ghosh and Gupta (43) found that around half of the covered beneficiaries under RSBY belonged to the non-poor category. This is not a healthy sign for the effective implementation of the schemes and it poses a challenge as a significant portion of benefits are siphoned off to better-off households (30, 44). This also represents the serious problem of mistargeting or the weak nature of targeting mechanisms in publicly financed health insurance schemes (45). State-level heterogeneity should also be considered, as coverage patterns and the scale of leakage differ substantially across states. Various studies reported interstate variation in RSBY enrolment among eligible beneficiaries, ranging from 14 percent in Delhi (46), to 68 percent in Karnataka (47) to 78 percent in Maharashtra (36).

## Conclusion

In the past decades, insurance coverage has shown marked improvement, particularly among the poor and vulnerable mainly through the GSHI schemes. The targeted intervention of health schemes like RSBY and other state health insurance schemes has shown considerable influence in the expansion of insurance coverage in India. However, the position of India remains in a dismal condition in providing health insurance to the people as compared to many developing countries. Being a developing country, India has a feasible option of the target-based health insurance scheme, but identifying the target population under GSHI is a major challenge in contemporary India due to a lack of time-bound and precise data and methodological issues of identification. The identification problem emerges from two sources, i.e., the exclusion of the targeted population and the inclusion of the non-targeted population. Despite the efforts of GSHI schemes, the majority of the poor households, i.e., the targeted population are not covered by any insurance scheme which partially emerged from a lack of awareness among the poor people. On the other hand, a significant share of the people in the middle and rich classes, i.e., non-targeted population are availing benefits of the GSHI mainly because of leakage problems. Thus, the exclusion of middle and rich classes and the inclusion of the poorest and poorer classes need to be rightsized in the implementation of existing GSHI like PM-JAY. Achieving universal health insurance coverage, particularly for the poor and vulnerable in India, will require concrete and evidence-linked strategies rather than broad implementation improvements alone. These include: linking the Socio-Economic and Caste Census (SECC) database with Aadhaar to improve beneficiary identification and reduce exclusion errors; conducting periodic (e.g., quarterly) enrolment audits to detect and reduce leakage to non-target groups; developing state-level targeting metrics to monitor inclusion and exclusion errors on an ongoing basis; and adopting community-based enrolment protocols that draw on local knowledge to identify eligible households missed by administrative lists. Complementary reforms like strengthening beneficiary identification systems more broadly, integrating state and central insurance programmes, expanding awareness campaigns, extending coverage to outpatient care, regulating private healthcare providers, improving quality assurance, reducing claim denials, and strengthening primary healthcare alongside insurance expansion would further support the goal of universal health insurance coverage.

### Limitations

There are several limitations in the study that should be noted. First, the health insurance status and its types are self-reported in NFHS, which is subject to recall bias. Second, each round of the NFHS is cross-sectional; while pooling three rounds allows temporal comparison, it does not support a strong causal interpretation of the trends observed. Third, the analysis is conducted at the national, pooled level and does not stratify by state, even though scheme design and implementation capacity vary considerably across states with their own supplementary insurance programmes. Fourth, the study examines coverage rather than downstream utilisation, quality of care, or health outcomes, which remain necessary complements to a full assessment of progress toward UHC. Finally, the BPL status and wealth quintiles used in the study serve as proxies for economic variables. However, these variables have certain limitations. For example, the BPL status may have targeting errors, exclude intended beneficiaries, and be inconsistent across states. Thus, having BPL cards does not necessarily mean one is economically poor. However, there is a relationship between these two variables (see Appendix Table 6).

## Data Availability

The study used secondary data from the International Institute for Population Sciences (IIPS) and ICF, which is publicly available at https://dhsprogram.com/countries/Country-Main.cfm?ctry_id=57, and with no access to personal identifiers.
