# Supplementary material for: Economic gradients of health insurance coverage in India
Source: Front Public Health. 2026 Aug 13;14:1872163. doi: 10.3389/fpubh.2026.1872163 (PMC13518565; doi:10.3389/fpubh.2026.1872163)
Supplement: Supplementary file 1 [file Supplementary_file_1.pdf]

Appendixes

Appendix Table 1: VIF of the different models applied in Table 3

Background characteristics	All pooled samples			NFHS-5
	Adjusted (M1)	Adjusted (M2)	Adjusted (M3)	Adjusted
**Survey rounds**				
2005-06 (Ref.)	-	-	-	-
2015-16	28.86	3.69		
2019-21	28.98	3.75		
**Wealth quintile**				
Poorest (Ref.)	-	-	-	-
Poorer	29.17	1.61		1.63
Middle	25.49	1.69		1.81
Richer	23.03	1.91		2.04
Richest	21.99	2.42		2.62
**Below Poverty Line**				
No (Ref.)	-	-	-	-
Yes	19.19	1.15		1.32
**Interaction between survey rounds and wealth quintile**				
2005-06 and Poorest (Ref.)	-	-	-	-
2015-16 and Poorer	16.91	-	-	-
2015-16 and Middle	14.7	-	-	-
2015-16 and Richer	13.07	-	-	-
2015-16 and Richest	12.12	-	-	-
2019-21 and Poorer	18.01	-	-	-
2019-21 and Middle	15.32	-	-	-
2019-21 and Richer	13.23	-	-	-
2019-21 and Richest	11.78	-	-	-
**Interaction between survey rounds and BPL**				
2005-06 and No BPL (Ref.)	-	-	-	-
2015-16 and BPL	12.63	-	-	-
2019-21 and BPL	15.61	-	-	-
**Place of residence**				
Urban (Ref.)	-	-	-	-
Rural	1.39	1.38	1.39	1.44
**Level of education**				
No Education Preschool (Ref.)	-	-	-	-
Primary	1.34	1.33	1.34	1.37
Secondary	1.59	1.59	1.59	1.61
Higher	1.48	1.48	1.48	1.48
**Caste**				
SC (Ref.)	-	-	-	-
ST	1.98	1.98	1.98	2.26
OBC	1.96	1.96	1.96	1.97
Others	2	2	2	2.01
**Religion**				
Hindu (Ref.)	-	-	-	-
Muslim	1.11	1.11	1.11	1.29
Christian	1.34	1.34	1.34	2.38
Others	1.07	1.07	1.07	1.56
Mean VIF	12.42	1.85	4.83	2.04

Note: VIF for the categories of economic variables from "Adjusted M3" is presented in Appendix Table 6

Appendix Table 2: VIF for the categories of economic variables (Adjusted M3)

Economic variables	VIF
**Interaction between survey rounds and wealth quintile**	
2005-06 and Poorest (Ref.)	-
2005-06 and Poorer	2.11
2005-06 and Middle	2.42
2005-06 and Richer	2.8
2005-06 and Richest	3.42
2015-16 and Poorest	10.21
2015-16 and Poorer	10.18
2015-16 and Middle	9.78
2015-16 and Richer	9.38
2015-16 and Richest	9.29
2019-21 and Poorest	11.18
2019-21 and Poorer	10.77
2019-21 and Middle	10.11
2019-21 and Richer	9.38
2019-21 and Richest	8.93
**Interaction between survey rounds and BPL**	
2005-06 and No BPL (Ref.)	-
2005-06 and BPL	1.37
2015-16 and No BPL	2.11
2019-21 and No BPL	1.73

Appendix Table 3: Percentage of households with health insurance coverage by social characteristics in India, 2005-06, 2015-16, and 2019-21

Variables	NFHS-3 (2005-06)	NFHS-4 (2015-16)	NFHS-5 (2019-21)
**Place of residence**			
Urban	10.4 (9.4, 11.4)	28.2 (27.6, 28.8)	38.1 (37.5, 38.7)
Rural	2.2 (2, 2.4)	28.9 (28.7, 29.2)	42.4 (42.2, 42.7)
**Level of education**			
No Education Preschool	1.3 (1.1, 1.5)	28.6 (28.2, 29)	41.2 (40.8, 41.5)
Primary	2.4 (2, 2.7)	30.2 (29.7, 30.7)	43.5 (43, 44)
Secondary	6.5 (6, 7)	27.3 (26.9, 27.7)	39.9 (39.6, 40.2)
Higher	18.7 (17.1, 20.3)	31.6 (30.7, 32.5)	40.5 (39.7, 41.3)
**Caste**			
SC	3.3 (2.9, 3.8)	31.1 (30.4, 31.8)	42.7 (42.1, 43.3)
ST	2.6 (2.1, 3.1)	30.8 (30, 31.6)	46.8 (46, 47.5)
OBC	3.8 (3.4, 4.2)	30.5 (30.1, 30.9)	43 (42.6, 43.4)
Others	7.7 (7.1, 8.4)	23.5 (22.9, 24)	34.6 (34, 35.1)
**Religion**			
Hindu	5.1 (4.7, 5.5)	29.8 (29.5, 30.2)	42.4 (42.1, 42.7)
Muslim	2.1 (1.7, 2.5)	20.1 (19.3, 20.8)	31 (30.1, 31.8)
Christian	7.3 (5.9, 8.7)	44.6 (43.1, 46.1)	55.3 (53.9, 56.7)
Others	7.9 (6.6, 9.3)	19.6 (18.6, 20.6)	31.3 (30.1, 32.5)
**Total**	4.9 (4.5, 5.3)	28.7 (28.3, 29)	41 (40.7, 41.3)

Source: National Family Health Surveys; Estimates are weighted applying the complex survey design

Appendix Table 4: Unadjusted and adjusted predicted prevalence of health insurance coverage by background characteristics in India, NFHS-3 (2005-06), NFHS-4 (2015-16), and NFHS-5 (2019-21)

Background characteristics	Pooled sample (NFHS-3, 4 and 5)		NFHS-5 (2019-21)	
	Unadjusted	Adjusted	Unadjusted	Adjusted
**Place of residence**				
Urban (Ref.)	31.4 (31, 31.8)	30 (29.6, 30.4)	38.1 (37.5, 38.7)	37.4 (37, 37.9)
Rural	33.2 (33, 33.4)	34 (33.7, 34.2)	42.4 (42.2, 42.7)	42.8 (42.6, 43.1)
**Level of education**				
No Education Preschool (Ref.)	31.5 (31.2, 31.7)	32.9 (32.6, 33.2)	41.2 (40.8, 41.5)	40.8 (40.4, 41.1)
Primary	34.3 (33.9, 34.6)	34.3 (34, 34.6)	43.5 (43, 44)	41.7 (41.3, 42)
Secondary	32 (31.7, 32.2)	31.1 (30.9, 31.4)	39.9 (39.6, 40.2)	40.3 (40, 40.6)
Higher	35.1 (34.5, 35.6)	34.3 (33.8, 34.9)	40.5 (39.7, 41.3)	43.1 (42.4, 43.8)
**Caste**				
SC (Ref.)	34.7 (34.2, 35.2)	34.1 (33.7, 34.5)	42.7 (42.1, 43.3)	43.2 (42.8, 43.7)
ST	36.5 (35.9, 37.1)	35.7 (35.2, 36.3)	46.8 (46, 47.5)	39.9 (39.3, 40.5)
OBC	34.3 (34, 34.6)	33.9 (33.7, 34.2)	43 (42.6, 43.4)	41.1 (40.8, 41.4)
Others	27.1 (26.7, 27.4)	28.3 (27.9, 28.6)	34.6 (34, 35.1)	39.4 (39, 39.9)
**Religion**				
Hindu (Ref.)	33.8 (33.6, 34)	33.5 (33.3, 33.8)	42.4 (42.1, 42.7)	41.7 (41.4, 42)
Muslim	23.7 (23.2, 24.3)	25.5 (24.9, 26)	31 (30.1, 31.8)	36.2 (35.5, 36.9)
Christian	46.8 (45.7, 47.8)	44.1 (43.2, 45.1)	55.3 (53.9, 56.7)	39.2 (38.1, 40.3)
Others	23.8 (23, 24.5)	24.1 (23.3, 24.9)	31.3 (30.1, 32.5)	42.7 (41.3, 44.2)

Note: The estimates are from the logistic regression models and the post-estimation of predicted probability from Table 3; the estimates are weighted by applying the complex survey design

Appendix Table 5: Unadjusted and adjusted predicted prevalence of health insurance coverage by states of India, NFHS-5 (2019-21)

States	Unadjusted	Adjusted
Jammu & Kashmir	13.8 (12.8, 14.9)	15 (13.8, 16.1)
Himachal Pradesh	38.9 (36.8, 41)	39.9 (37.8, 42)
Punjab	25.2 (24.1, 26.2)	24.1 (22.7, 25.4)
Chandigarh	32.2 (25.9, 38.5)	36.7 (30.5, 43)
Uttarakhand	62.5 (60.6, 64.3)	63.2 (61.4, 65)
Haryana	25.7 (24.6, 26.7)	25.6 (24.5, 26.6)
NCT of Delhi	25 (22.9, 27.1)	29.4 (27.1, 31.7)
Rajasthan	87.8 (87, 88.6)	88.4 (87.6, 89.2)
Uttar Pradesh	15.9 (15.4, 16.3)	16.7 (16.2, 17.2)
Bihar	17.4 (16.7, 18.1)	17.1 (16.4, 17.7)
Sikkim	28 (24.7, 31.4)	28.8 (25.3, 32.3)
Arunachal Pradesh	29.3 (27.8, 30.8)	28.7 (27, 30.4)
Nagaland	22 (20.1, 24)	23.2 (21, 25.4)
Manipur	16.4 (14.9, 17.8)	18.2 (16.6, 19.8)
Mizoram	50.3 (47.7, 52.8)	57 (54, 60)
Tripura	36.4 (34.4, 38.5)	38.4 (36.4, 40.5)
Meghalaya	69 (66.5, 71.6)	71.1 (68.6, 73.7)
Assam	66.7 (65.6, 67.7)	69.1 (68, 70.1)
West Bengal	33.7 (32.4, 35)	36.8 (35.5, 38.1)
Jharkhand	50.3 (49, 51.6)	49.9 (48.7, 51.2)
Odisha	47.9 (46.9, 49)	47.4 (46.3, 48.5)
Chhattisgarh	71.4 (69.9, 72.8)	65.5 (63.9, 67)
Madhya Pradesh	38.1 (37.3, 38.9)	36.9 (36.1, 37.7)
Gujarat	44.4 (43.4, 45.4)	46.1 (45.1, 47.1)
Dadra And Nagar Haveli and Daman & Diu	56.6 (52.6, 60.6)	59.7 (56, 63.5)
Maharashtra	22.4 (21.3, 23.5)	23.5 (22.5, 24.6)
Andhra Pradesh	80.2 (79.1, 81.2)	74.6 (73.3, 75.8)
Karnataka	31.8 (30.5, 33.1)	27.3 (26.1, 28.5)
Goa	73.1 (70, 76.1)	74.8 (71.8, 77.7)
Lakshadweep	67.7 (61.7, 73.7)	75.4 (70.3, 80.6)
Kerala	57.8 (56.4, 59.2)	58.2 (56.9, 59.6)
Tamil Nadu	66.5 (65.5, 67.5)	68.2 (67.2, 69.2)
Puducherry	30.1 (22.7, 37.6)	32.1 (24.6, 39.6)
Andaman & Nicobar Islands	1.8 (1.2, 2.4)	2.4 (1.6, 3.3)
Telangana	69.2 (68.2, 70.1)	62.6 (61.5, 63.7)
Ladakh	17.1 (14.4, 19.7)	17.8 (15, 20.7)

Note: The estimates are from the logistic regression models and the post-estimation of predicted probability from Table 3; the estimates are weighted by applying the complex survey design

Appendix Table 6: Percentage of BPL households across different wealth quintiles in India, 2005-06, 2015-16, and 2019-21

Wealth quintiles	2005-06	2015-16	2019-21	Pooled Sample
Poorest	39 (37.6, 40.5)	54.7 (54.2, 55.2)	60 (59.5, 60.5)	56 (55.6, 56.3)
Poorer	37.2 (36, 38.5)	49.1 (48.6, 49.7)	55.1 (54.6, 55.6)	51 (50.7, 51.3)
Middle	31.5 (30.3, 32.7)	42.9 (42.2, 43.5)	50.2 (49.6, 50.8)	45.4 (45.1, 45.8)
Richer	21.4 (20.3, 22.5)	32 (31.4, 32.6)	39.6 (39, 40.2)	34.7 (34.3, 35.1)
Richest	7.1 (6.4, 7.7)	14.4 (13.7, 15)	19.3 (18.8, 19.9)	16.1 (15.7, 16.5)

Note: Complex survey design weight is applied

****
